# High incidence of fractures after R-CHOP-like chemotherapy for aggressive B-cell non-Hodgkin lymphomas

**DOI:** 10.1007/s00520-021-06120-0

**Published:** 2021-03-10

**Authors:** Li-Wen Huang, Dong Sun, Thomas M. Link, Thomas Lang, Weiyun Ai, Lawrence D. Kaplan, Michael A. Steinman, Charalambos Andreadis

**Affiliations:** 1 Helen Diller Family Comprehensive Cancer Center, University of California San Francisco, San Francisco, CA USA; 2 Division of Hematology/Oncology, Department of Medicine, San Francisco Veterans Affairs Medical Center, San Francisco, CA USA; 3 Musculoskeletal Quantitative Imaging Research Group, Department of Radiology and Biomedical Imaging, University of California San Francisco, San Francisco, CA USA; 4 Department of Radiology, Tongji Hospital, Tongji Medical College, Huazhong University of Science and Technology, Wuhan, China; 5 School of Dentistry, University of California, San Francisco, CA USA; 6 Division of Geriatrics, Department of Medicine, San Francisco Veterans Affairs Medical Center, San Francisco, CA USA

**Keywords:** Non-Hodgkin lymphoma, Fractures, Bone health, RCHOP

## Abstract

**Purpose:**

Patients with non-Hodgkin lymphoma (NHL) have a median age of 67, with 70% surviving over 5 years. Chemotherapy for aggressive NHL includes cyclophosphamide, anthracycline, and high doses of corticosteroids, which can impair bone health. By reviewing clinical characteristics and standard-of-care CT scans, we evaluate the prevalence and incidence of fractures and the clinical correlates of fractures in patients treated for aggressive B-cell NHL.

**Methods:**

We retrospectively reviewed patients seen at the University of California San Francisco lymphoma clinic from January 1, 2016, to March 31, 2017 who had (1) aggressive B-cell NHL, (2) received first-line therapy with R-CHOP-like regimens, and had (3) CT scans pre- and post-treatment available for review. Associations between clinical variables and vertebral, rib, and pelvic fracture outcomes were assessed, and multivariate logistic regression models were used to identify predictors of prevalent and incident fractures.

**Results:**

We identified 162 patients who met the inclusion criteria. Median age at diagnosis was 60 years. Of the 162 patients, 38 patients (28%) had prevalent fractures prior to receiving chemotherapy. Within 1 year after treatment, 16 patients (10%) developed new fractures. Having a prevalent fracture strongly predicted developing a new fracture after treatment, with incident fractures occurring in 12 of 38 patients with prevalent fractures versus 4 of 124 without prevalent fractures (odds ratio 10.45, *p*<0.0005).

**Conclusion:**

Our results suggest that patients with aggressive B-cell NHL who receive R-CHOP-like therapy should be screened for fractures prior to treatment and those with existing fractures should be considered for therapy to decrease risk of new fractures.

## Introduction

Non-Hodgkin lymphoma (NHL) is the seventh most common cancer diagnosis in the USA, and there are an estimated 719,831 people living with NHL in the USA. The advancement of effective treatments for NHL has resulted in a high proportion of patients achieving long-term survival, with about 72.7% of patients surviving over 5 years [1]. Thus, there is an increasing need for improved awareness and management of long-term complications experienced by survivors, such as impaired bone health. Deficits in skeletal health are known side effects of the most common treatment regimen for aggressive NHL, which is immunochemotherapy with R-CHOP (rituximab, cyclophosphamide, doxorubicin, vincristine, prednisone) or an R-CHOP-like regimen [2]. Cyclophosphamide, anthracycline, and the high doses of corticosteroids used in R-CHOP can result in significant bone loss [3–6]. Furthermore, vincristine can cause peripheral neuropathy, which can increase the risk of falls and thus the risk of fracture in someone with osteoporosis [7, 8]. Bone health and fracture risk are particularly important in older patients, who comprise the majority of patients with NHL, who have a median age at diagnosis of 67 years [1]. Older age is a strong predictor of osteoporosis, and fractures in older adults can cause significant morbidity and mortality [9–12].

The clinical characteristics associated with a higher risk of fractures in NHL patients are not well characterized. A few studies have found that NHL patients experience reduced bone mineral density (BMD) and a higher risk for fractures after treatment compared to baseline [13–15]. These studies identified different predictors of reduced BMD or fractures, including older age [13] and lower baseline vertebral density [15] predicting a higher risk of fractures and different lab parameters predicting BMD loss in different locations [14]. While low BMD in itself is not necessarily symptomatic or detrimental to physical health, fractures can result in morbidity and decreased quality of life. Although low BMD is associated with fracture risk, there is wide overlap in the BMD of patients who develop a fracture and people who do not [16], and the fracture risk for a given dual energy x-ray absorptiometry T-score varies significantly with age [17].

Thus, our goal in this study was to achieve a better understanding of the clinical factors associated with higher fracture risk to help guide the supportive care of bone health in patients treated for aggressive NHL. In this single-center retrospective study, we used CT scans obtained as a part of standard routine care for the treatment of lymphoma to (1) describe the prevalence and incidence of fractures and (2) identify risk factors for increased fractures in patients treated for aggressive B-cell NHL.

## Materials and methods

### Patient population

This is a retrospective study of patients seen in the University of California San Francisco lymphoma clinic from January 1, 2016, to March 31, 2017. Patients were included if they met the following inclusion criteria: (1) histologically confirmed aggressive B-cell NHL including diffuse large B-cell lymphoma, high-grade B cell lymphoma, and primary mediastinal B-cell lymphoma; (2) receipt of first-line therapy with R-CHOP or R-CHOP-like regimens (e.g., R-EPOCH, R-CODOX-M); and (3) availability of CT scans of the chest, abdomen, and pelvis pre- and post-first-line therapy for radiology review. Patients could be diagnosed and treated before the study chart review time period (from January 1, 2016, to March 31, 2017) as long as their CT scans pre- and post-first-line therapy were available for review.

Baseline clinical characteristics (e.g., stage, International Prognostic Index (IPI) score, baseline lactate dehydrogenase (LDH)), existing diagnosis of osteoporosis on patient’s problem list, documentation of traumatic fractures, and information about baseline use of bone-active medications (i.e., corticosteroids, calcium, vitamin D, and bisphosphonates) were retrieved from medical records when available. Treatment information (number of chemotherapy cycles, radiation therapy, treatment response) was also collected from medical records.

### CT studies and fracture assessment

Pre-treatment CT or PET/CT scans were performed in the routine staging evaluation for aggressive B-cell NHL, and post-treatment scans were performed as part of standard of care treatment evaluation and monitoring. Pre-treatment CT images had to be obtained before initiation of first-line therapy. Post-first-line therapy scans could be obtained at the end of therapy (typically about 5 months after treatment initiation) or up to 1-year post-treatment. In cases where more than one scan was available for review, the scan that was closer to 1 year was preferentially selected for review. The CT studies could be performed as stand-alone CTs or as part of a PET/CT.

Axial, sagittal, and coronal reconstructions of chest, abdomen, and pelvis CT images were reviewed and graded by a radiologist (DS) to identify prevalent and incident fractures of the vertebrae, ribs, and pelvis. As shown by a previous study, sagittal CT reconstructions are more accurate than standard spine radiographs in assessing vertebral fracture deformity [18]. CT is also a standard technique to detect rib and pelvic fractures and more sensitive than standard radiographs [19, 20]. Cases identified as fractures were confirmed by a second radiologist (TML). Vertebral fractures were graded as grades 1, 2, or 3 according to the Genant criteria [21]. Per the Genant criteria, progression of an existing vertebral fracture to a higher grade was counted as an incident fracture. Documented traumatic fractures were not included. We did not have information about whether the identified fractures were symptomatic. However, the occurrence of any non-traumatic fracture or fracture resulting from minor trauma (such as fall from standing height) is sufficient for a clinical diagnosis of osteoporosis. Since most of these fractures are presumably non-traumatic, these fractures are considered clinically significant.

### Statistical analysis

Descriptive statistics such as medians and frequency were used to summarize demographic and clinical characteristics. Patients were grouped by their fracture status at baseline, and the association between clinical characteristics and prevalent fractures was assessed using univariate logistic regression. The patients were then grouped by occurrence of new fractures after treatment, and associations between clinical characteristics and incident fractures were analyzed similarly. Multivariate logistic regression models were used to identify predictors of prevalent and incident fractures, adjusting for clinical variables that were significant in univariate analysis with a two-sided *p*-value of less than 0.05. When clinical variables that were significant in univariate analysis were not independent from each other (specifically, the IPI score with any of its components), we did not analyze them together in a multivariate logistic regression model. Instead, available components of the IPI score (age, stage, LDH) were included in a multivariate logistic regression model. We did not have information about the remaining two components of the IPI score (performance status, extranodal sites). All statistical analyses were performed using STATA (version 15.1, College Station, Texas, USA).

## Results

### Clinical and treatment characteristics

In total, 162 patients were included. The median age at diagnosis was 60 years old (range 19–87 years). Of the sample, 39% were female. The majority of patients had diffuse large B-cell lymphoma (89%), which is the most common type of aggressive NHL. By Ann Arbor Staging, 20% were stage 1, 26% stage 2, 18% stage 3, and 36% stage 4. By the IPI score, 38% were considered low risk, 28% low-intermediate risk, 14% high-intermediate risk, and 15% high risk (5% missing IPI information). Most patients received treatment with R-CHOP (60%) or R-EPOCH (37%). Only 15% also received radiation therapy.

Only 5 (3.2%) patients had a known diagnosis of osteoporosis based on chart review at the time of NHL diagnosis. At baseline, 20 patients (12.3%) were taking calcium, 24 (14.8%) were taking vitamin D, and 3 (1.9%) were receiving bisphosphonates. Twelve patients (7.4%) had a course of steroids before starting treatment for their lymphoma, as is often done while awaiting treatment initiation in particularly symptomatic patients. Other baseline clinical and treatment characteristics are summarized in Table 1.

**Table 1:** Clinical characteristics (*n*=162)

Clinical characteristic	Results
Age in years, median (range)	60 (19–87)
Female, *n* (%)	63 (39%)
BMI (kg/m^2^), mean (SD)	26 (5.4)
Diagnosis, *n* (%)	
• DLBCL	144 (89%)
• PMBCL	9 (5.5%)
• High-grade BCL	9 (5.5%)
Ann Arbor Stage, *n* (%)	
• Stage I	33 (20%)
• Stage II	42 (26%)
• Stage III	29 (18%)
• Stage IV	58 (36%)
International Prognostic Index score, *n* (%)	
• 0–1 (low risk)	61 (38%)
• 2 (low-intermediate risk)	45 (28%)
• 3 (high-intermediate risk)	23 (14%)
• 4–5 (high risk)	24 (15%)
• Missing	9 (5%)
Bone marrow involvement, *n* (%)	31 (20%)
Prior diagnosis of osteoporosis, *n* (%)	5 (3%)
Bone active medications, *n* (%)	
• Vitamin D	24 (15%)
• Calcium	20 (12%)
• Bisphosphonate	3 (2%)
First-line therapy regimen, *n* (%)	
• R-CHOP	97 (60%)
• R-EPOCH	60 (37%)
• Other	5 (3%)
Number of cycles, median (range)	6 (2–8)
Radiation therapy, *n* (%)	25 (15%)
Response to first-line therapy, *n* (%)	
• Complete response	141 (87%)
• Partial response	11 (7%)
• Progressive disease	10 (6%)

*BMI* body mass index; *SD* standard deviation; *DLBCL* diffuse large B-cell lymphoma; *PMBCL* primary mediastinal large B-cell lymphoma; *BCL* B-cell lymphoma; *R-CHOP* rituximab, cyclophosphamide, doxorubicin, vincristine, prednisone; *R-EPOCH* rituximab, etoposide, prednisone, vincristine, cyclophosphamide, doxorubicin

### Prevalent fractures

**Fig. 1:**
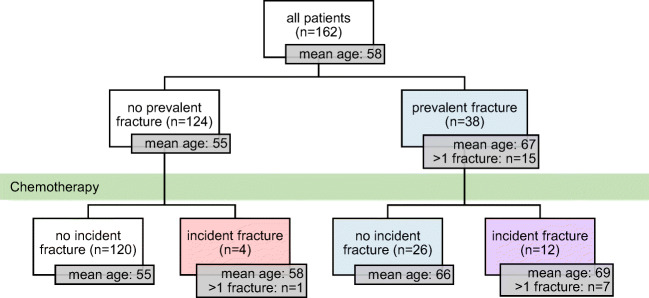
Distribution of prevalent and incident fractures

At baseline, 38 of 162 patients (23%) had a vertebral, rib, or pelvic fracture identified on their pre-treatment CT scan, and 18 patients had moderate or severe (Genant grade 2–3) vertebral fractures. Of these 38, 15 patients (39%) had more than one identified fracture, including 2 patients with 8 fractures each (6 vertebral + 2 rib, 2 vertebral + 6 rib) (Fig. 1). Only 2 of these 38 had a diagnosis of osteoporosis noted on their medical chart at the time of initial evaluation.

Associations between clinical factors and prevalent fracture are shown in Table 2. In unadjusted analysis, older patients had a higher risk of having a baseline fracture (odds ratio (OR) 1.06 per year of age, 95% confidence interval [CI] 1.03–1.10, *p*<0.0005). Patients with an IPI score >1 indicating intermediate or high-risk disease were also more likely to have a prevalent fracture than those with a low-risk IPI score (OR 2.9, 95% CI 1.22–6.90, *p*=0.02). The following baseline clinical characteristics were not significantly associated with prevalent fractures: sex, BMI, diagnosis, stage, LDH, bone marrow involvement, prior diagnosis of osteoporosis, and bone active medication use. In multivariate analysis adjusting for the available components of the IPI score (age, stage, LDH), age was the only variable significantly associated with prevalent fracture (OR 1.06 per year of age, 95% CI 1.03–1.10, *p*<0.0005; Table 2).

**Table 2:** Associations between clinical factors and prevalent fracture

			Univariate analysis		Multivariate analysis*	
Clinical characteristic	Prevalent fracture (*n*=38)	No prevalent fracture (*n*=124)	OR (95% CI)	*p*-value	OR (95% CI)	*p*-value
Age in years, mean ± SD	67 ± 14.6	55 ± 14.6	*1.06 (1.03–1.10)*	*<0.0005*	*1.06 (1.03–1.10)*	*<0.0005*
Female, *n* (%)	14 (37)	49 (40)	1.12 (0.53–2.37)	0.77		
BMI (kg/m^2^), mean ± SD	26 ± 4.5	26 ± 5.6	1.01 (0.94–1.09)	0.82		
Diagnosis, *n* (%)						
• DLBCL	32 (84)	112 (90)	ref.			
• Other	6 (16)	12 (10)	1.75 (0.61–5.03)	0.30		
Ann Arbor Stage, *n* (%)						
• Stage I–II	16 (42)	59 (48)	ref.		ref.	
•Stage III–IV	22 (58)	65 (52)	1.25 (0.60–2.60)	0.55	1.19 (0.53–2.68)	0.68
IPI score, *n* (%)						
• 0–1 (low risk)	8 (22)	53 (45)	ref.			
• 2–5 (intermediate to high risk)	28 (78)	64 (55)	*2.90 (1.22–6.90)*	*0.02*		
LDH, mean ± SD	334 ± 257	398 ± 965	1.00 (1.00–1.00)	0.71	1.00 (1.00–1.00)	0.77
Bone marrow involvement, *n* (%)	8 (22)	23 (19)	1.18 (0.48–2.91)	0.73		
Prior diagnosis of osteoporosis, *n* (%)	2 (5)	3 (3)	2.23 (0.36–13.87)	0.39		
Bone active medications, *n* (%)						
• Vitamin D	4 (11)	20 (16)	0.61 (0.20–1.92)	0.40		
• Calcium	5 (13)	15 (12)	1.10 (0.37–3.26)	0.86		
• Bisphosphonate	1 (3)	2 (2)	1.65 (0.15–18.70)	0.69		

*OR* odds ratio, *CI* confidence interval, *SD* standard deviation, *BMI* body mass index, *DLBCL* diffuse large B-cell lymphoma, *IPI* International Prognostic Index, *LDH* lactate dehydrogenase

*Because the variables significant at *p*<0.05 in univariate analysis (age and IPI) are not independent, multivariate logistic regression was performed with the available components of the IPI score (age, stage, LDH) instead

Statistically significant ORs with p-value <0.05 are italicized for emphasis

### Incident fractures

Within 1 year after treatment with R-CHOP-like therapy, 16 of 162 patients (10%) had a new vertebral, rib, or pelvic fracture identified on their post-treatment CT scan. Focusing on vertebral fractures, 11 patients (7%) developed new vertebral fractures, 8 of whom had new moderate or severe vertebral fractures. Of these 16, 7 (44%) had more than 1 new fracture, including 1 patient with 7 new rib fractures (Fig. 1). Those who had a fracture at baseline were more likely to develop a new fracture (OR 13.85, 95% CI 4.14–46.36, *p*<0.0005). Only 1 of these 16 had a diagnosis of osteoporosis noted on their medical chart at the time of initial evaluation. None of the incident fractures were associated with the site of radiotherapy, and one patient developed an incident fracture at a site of tumor involvement.

Associations between clinical factors and incident fracture are shown in Table 3. In unadjusted analysis, older patients had a higher risk of developing a new fracture (OR 1.05 per year of age, 95% CI 1.01–1.09, *p*=0.02). Patients who received fewer cycles of first-line chemotherapy had a lower risk of incident fracture (OR 0.60, 95% CI 0.38–0.94, *p*=0.03). The following clinical and treatment characteristics were not significantly associated with incident fractures: sex, BMI, diagnosis, stage, IPI, LDH, bone marrow involvement, prior diagnosis of osteoporosis, first-line chemotherapy regimen, receipt of radiation, and achievement of complete remission at the end of first-line therapy. The median follow-up time between scans was similar between those with and without incident fracture (10.5 and 11 months, respectively).

**Table 3:** Associations between clinical factors and incident fracture

			Univariate analysis		Multivariate analysis*	
Clinical characteristic	Incident fracture (*n*=16)	No incident fracture (*n*=146)	OR (95% CI)	*p*-value	OR (95% CI)	*p*-value
Age in years, mean ± SD	67 ± 11.4	57 ± 15.5	*1.05 (1.01–1.09)*	*0.02*	1.02 (0.98–1.07)	0.29
Female, *n* (%)	6 (38)	57 (39)	1.07 (0.37–3.10)	0.90		
BMI (kg/m^2^), mean ± SD	26 ± 4.5	26 ± 5.6	0.97 (0.86–1.10)	0.65		
Diagnosis, *n* (%)						
• DLBCL	13 (81)	131 (90)	ref.			
• Other	3 (19)	15 (10)	2.02 (0.52–7.89)	0.31		
Ann Arbor Stage, *n* (%)						
• Stage I–II	6 (38)	69 (47)	ref.			
• Stage III–IV	10 (63)	77 (53)	1.49 (0.52–4.32)	0.46		
IPI score, *n* (%)						
• 0–1 (low risk)	3 (20)	58 (42)	ref.			
• 2–5 (intermediate to high risk)	12 (80)	80 (58)	2.90 (0.78–10.74)	0.11		
LDH, mean ± SD	378 ± 285	384 ± 893	1.00 (1.00–1.00)	0.98		
Bone marrow involvement, *n* (%)	3 (19)	28 (20)	0.94 (0.25–3.52)	0.93		
Prior diagnosis of osteoporosis, *n* (%)	1 (6)	4 (3)	2.28 (0.24–21.78)	0.47		
Bone active medications, *n* (%)						
• Vitamin D	3 (19)	21 (14)	1.37 (0.36–5.23)	0.64		
• Calcium	2 (13)	18 (12)	1.02 (0.21–4.84)	0.98		
• Bisphosphonate	0 (0)	3 (2)	--^†^	--		
Prevalent fracture, *n* (%)	12 (75)	26 (18)	*13.85* *(4.14–46.36)*	*<0.0005*	*10.45* *(2.90–37.74)*	*<0.0005*
First-line therapy regimen, *n* (%)						
• R-CHOP	9 (56)	88 (60)	ref.			
• R-EPOCH	6 (38)	54 (37)	1.09 (0.37–3.22)	0.88		
• Other	1 (6)	4 (3)	2.44 (0.25–24.29)	0.45		
Number of cycles, mean ± SD	5.3 ± 1.3	5.8 ± 0.8	*0.60 (0.38–0.94)*	*0.03*	0.62 (0.37–1.04)	0.07
Radiation therapy, *n* (%)	3 (19)	22 (15)	1.29 (0.34–4.90)	0.71		
CR after first-line therapy, *n* (%)	12 (75)	129 (88)	0.40 (0.11–1.37)	0.14		

*OR* odds ratio; *CI* confidence interval; *SD* standard deviation; *BMI* body mass index; *DLBCL* diffuse large B-cell lymphoma; *IPI* International Prognostic Index; *LDH* lactate dehydrogenase; *R-CHOP* rituximab, cyclophosphamide, doxorubicin, vincristine, prednisone; *R-EPOCH* rituximab, etoposide, prednisone, vincristine, cyclophosphamide, doxorubicin; *CR* complete remission

*Multivariate logistic regression performed with variables significant at *p*<0.05 in univariate analysis

^†^Bisphosphonate use predicted no incident fracture perfectly, so it was dropped from the univariate logistic regression

Statistically significant ORs with p-value <0.05 are italicized for emphasis

In a multivariate logistic regression model including prevalent fracture, age, and cycles received, prevalent fracture was strongly predictive of incident fracture with an OR of 10.45 (95% CI 2.90–37.74, *p*<0.0005) (Table 3). In multivariate analysis, age and cycles received were no longer statistically significantly associated with the development of incident fracture.

### Fracture characteristics

Fracture burden and distribution are summarized in Table 4. High fracture burden with more than one fracture was seen in a substantial portion of patients with fractures either at baseline (39%) or after treatment (44%). The distribution of fracture sites differed between prevalent and incident fractures. Eighty-two percent of those with prevalent fractures had vertebral-only fractures compared to 56% of those with incident fractures, while 3% had prevalent rib-only fractures compared to 25% incident rib-only fractures. Table 5 details information about fracture location and grade for individuals with incident fractures. Only patients with any prevalent fractures developed moderate or severe (Genant grade 2–3) incident vertebral fractures.

**Table 4:** Fracture characteristics

	Prevalent fracture	Incident fracture
Number of patients	38	16
• Number with prevalent fracture	--	12 (75%)
• Number with >1 fracture	15 (39%)	7 (44%)
Fracture sites		
• Vertebral only	31 (82%)	9 (56%)
• Rib only	1 (3%)	4 (25%)
• Pelvic only	1 (3%)	1 (6%)
• Vertebral + rib	4 (11%)	1 (6%)
• Vertebral + pelvic	0	0
• Rib + pelvic	1 (3%)	0
• Vertebral, rib, pelvic	0	1 (6%)

**Table 5:** Fracture location and grade in individuals with incident fractures

Age/sex	# Prevalent fracture	Prevalent fracture location (Genant grade if vertebral)	# Incident fracture	Incident fracture location (Genant grade if vertebral)
47M	None	--	1	T9 (1)
55F	None	--	3	Ribs right 5–7
58M	None	--	1	Rib left 12
73M	None	--	1	T11 (1)
62M	1	L2 (1)	1	T12 (1)
72F	1	L1 (3)	2	Ribs right 9–10
73F	1	L3 (3)	1	L1 (3)
80M	1	Rib right 3	3	L1 (2), L4 (3), L5 (1)
81M	1	L1 (3)	1	L4 (2)
85F	1	Pelvic	1	Pelvic
55M	2	L2 (2), L3 (2)	2	L1 (1), L2 (3)
76F	2	L1 (2), L2 (1)	1	L2 (2)
57M	3	T11 (1), L2 (2), L3 (1)	7	Ribs right 6–8, 10–11, ribs left 7–8
55F	5	Ribs right 1, 3, 11, rib left 12, pelvic	2	L2 (1), rib right 12
58F	5	T12 (1), ribs right 4, 6–8	1	T12 (3)
65M	8	T6 (1), T7 (1), T11 (1), T12 (1), L2 (2), L3 (3), rib left 8, rib right 5	5	T5 (3), T6 (3), rib left 6, rib right 4, pelvic

## Discussion

In this study of 162 patients newly diagnosed with aggressive B-cell NHL and treated with R-CHOP-like chemotherapy, we found that 10% of patients developed a new vertebral, rib, or pelvic fracture within 1 year after therapy. This far exceeds what may be expected in the general population based on reported fracture risk in large cohort studies. For example, women aged 65–69 who do not have a baseline fracture have an annual vertebral fracture incidence of 0.5% (compared to 7% in our study), and women with baseline vertebral deformities have a 5.4-fold increased risk of new vertebral deformities and 1.9-fold increased risk of any nonvertebral fractures [22, 23]. Our study adds to the literature of the adverse effects on bone health of R-CHOP-like therapy for lymphomas by describing in more detail the characteristics of these fractures and evaluating potential predictors of increased fracture risk. Having a fracture at baseline was an extremely strong predictor of developing a new fracture after treatment, with incident fractures occurring in 12 of 38 patients with prevalent fractures compared to 4 of 124 patients without prevalent fractures.

Our finding of 10% incident vertebral, rib, or pelvic fractures is similar to reported rates ranging 12.5–14% in prior studies with smaller numbers of lymphoma patients, though each study evaluated slightly different types of fractures. In a single-center study of 32 patients with a median age of 59 years with any lymphoma (majority DLBCL) treated by chemotherapy, Paccou et al. reported significantly decreased BMD at 1 year after chemotherapy compared to baseline; in this study, 4 patients (12.5%) developed new symptomatic osteoporotic vertebral or rib fractures during 1 year of follow-up [14]. In another study with 111 patients with DLBCL treated with R-CHOP-like regimens, Svendsen et al. found that vertebral density was significantly reduced for up to 2 years after treatment, and vertebral compression fractures were visualized on CT in 16 (14%) patients during follow-up [15]. Our study also provides additional support for age as an important risk factor for fractures in patients with aggressive B-cell NHL. In one prior study, Cabanillas et al. used SEER-Medicare data to evaluate rates of any fracture and osteoporosis in elderly NHL patients and found that NHL patients who received chemotherapy had significantly higher rates of osteoporosis and any fracture than those who did not receive chemotherapy [13]. Older age was not found to be associated with greater BMD decline in the other two studies [14, 15], although this may be due to insufficient power.

More importantly, our study identifies prevalent fracture before treatment as a novel risk factor that strongly predicts new fractures in patients with aggressive B-cell NHL with an OR of 10.45. The prevalent vertebral, rib, or pelvic fracture rate of 23% seen in this study even prior to receiving any treatment exceeds the reported prior clinical fracture rate of 17.6% in women with a mean age of 64.9 years and 8.7% in men with a mean age of 64.5 years in a large population-based study [12]. Notably these population comparisons are older than our study population (median age 60) and thus would be expected to have a higher rate of fractures. Interestingly, studies from the pediatric acute lymphoblastic leukemia literature have also made similar observations of high fracture burden prior to therapy. In children newly diagnosed with acute lymphoblastic leukemia, 16% of patients were found to have prevalent vertebral compression fractures, and 48% of those with fractures had multiple fractures (compared to 39% in our study) [24]. After treatment, 16% of patients developed new fractures, and 52% of these were noted to have fractures at baseline. Having a vertebral fracture at baseline was associated with incident fracture at 12 months by an OR of 7.3 [25].

These observations suggest that there are two processes at play here, one process intrinsic to a lymphoma diagnosis and a second extrinsic process related to lymphoma-directed therapy. The high prevalence of fractures before any therapy suggests that some aspect of the disease biology of lymphoma compromises bone health. On top of this intrinsic process, R-CHOP-like chemotherapy may further impair bone health and ultimately result in a higher incidence of fractures. While our study is not designed to differentiate between the effects of lymphoma versus chemotherapy on bone health, existing studies suggest that chemotherapy for lymphoma impairs bone health independent of the lymphoma diagnosis. One recent national cohort study found that patients treated for DLBCL and follicular lymphoma were at higher risk of osteoporotic events compared to the matched general population, while a sensitivity analysis showed that follicular lymphoma patients managed with a watch-and-wait strategy for at least a year had a similar risk of osteoporotic events compared to the matched population, suggesting that the lymphoma treatment is what leads to an increased risk of osteoporotic events [26]. Part of the mechanism by which R-CHOP-like chemotherapy results in fracture may be an acceleration of bone loss beyond what is expected with normal aging, which has been shown in other studies [14, 15]. In addition, the disproportionately higher number of rib fractures after chemotherapy raises the possibility of increased fractures from falls, which patients may be at risk of if they develop sarcopenia or vincristine-related peripheral neuropathy [27, 28]. However, since we do not have information on skeletal muscle density, peripheral neuropathy, or falls, this possibility is speculative.

The association between new fractures and receipt of fewer cycles of first-line chemotherapy in unadjusted analysis was unexpected, and we performed exploratory analyses to better understand this association. We explored potential confounders for number of cycles received and fractures, such as age (as a potential surrogate for frailty), chemotherapy regimen (as a surrogate for treatment intensity), and remission status (since patients who do not achieve complete remission may receive fewer cycles before proceeding to salvage chemotherapy and autologous stem cell transplantation, which may further impair bone health). Complete remission after frontline therapy was associated with a greater number of cycles, while receipt of a regimen other than R-CHOP or R-EPOCH was associated with a smaller number of cycles; age was not associated with number of cycles. In exploratory multivariate analysis adjusting for complete remission after first-line therapy and chemotherapy regimen, the number of cycles of first-line chemotherapy received was no longer significantly associated with new fractures.

While age and existing fractures are known to be risk factors for new fractures in the general population [29, 30], they have historically not been recognized as risk factors for new fractures in adults with aggressive B-cell NHL. If replicated, our findings may have important implications for targeting treatment to the highest risk patients. While calcium, vitamin D, and bisphosphonates may be reasonable to consider across the board, we should strongly consider them in the patients who have prevalent fractures on their baseline staging CT scans. Guidelines from the American College of Rheumatology recommend calcium and vitamin D for anyone taking ≥2.5 mg/day of prednisone for ≥3 months. For adults ≥30 years of age who are receiving very high-dose glucocorticoid (initial prednisone dose of ≥30 mg/day and a cumulative annual dose of >5 gm), initiation of oral bisphosphonate is recommended [31]. R-CHOP, given at 21-day intervals for typically 6 cycles, is completed in a little over 4 months. With each cycle, the prednisone dose is typically around 100 mg/day for days 1 through 5 [32], resulting in a cumulative dose of 3 gm in a little over 4 months, which averages out to 24 mg/day. Thus, one can argue that patients undergoing R-CHOP-like therapy should at least be recommended to start calcium and vitamin D based on average daily dose of prednisone ≥2.5 mg/day for ≥3 months and possibly even be considered for oral bisphosphonate therapy based on the high-doses received (if 3 gm in 4 months is considered at least as bad as 5 gm in 1 year). The American College of Rheumatology also recommends oral bisphosphonate therapy for adults ≥40 years of age who are at moderate risk of major fracture, with moderate risk defined as 10-year risk of major osteoporotic fracture of 10–19% based on the glucocorticoid-adjusted fracture risk assessment tool (FRAX) (https://www.sheffield.ac.uk/FRAX/tool.aspx). The 10–14% risk of fractures in 1–2 years seen in our study and previous studies in similar populations would be similarly clinically compelling.

Current National Comprehensive Cancer Network recommendations for bone health for patients with B-cell lymphomas who received steroid-containing regimens recommend post-treatment BMD evaluation but not until 1 year after therapy [2]. However, insights from rheumatologic studies show that the highest rate of bone loss with glucocorticoid therapy occurs within the first 3–6 months of therapy [31]. This combined with the high rates of fracture in less than a year shown in our and similar studies suggests that the current recommendation for bone health evaluation 1 year after therapy may be too late, and preventative measures at treatment initiation should be considered in patients who are at highest risk for developing fractures. Importantly, bone architecture changes irreversibly with bone loss, so a preventative strategy is superior to a restorative strategy since growth of new bone will only occur on existing bone as a template [33]. In fact, small studies have shown that the use of bisphosphonates in patients with newly diagnosed lymphoma receiving primary treatment can prevent bone loss and reduce the risk of fractures [34, 35]. Our study takes the first step in identifying those who may be at highest risk and thus are candidates for preventative measures.

Limitations of this study include its retrospective nature and the lack of a non-cancer or a non-chemotherapy control group, and we are unable to determine if the high fracture rates are associated with a lymphoma-related or chemotherapy-related process; thus the results are descriptive and hypothesis-generating. Importantly, the small number of fracture events means we do not have sufficient power to adjust for all potential confounders in multivariate analyses, and thus multivariate analyses were performed with only a limited number of predictors. We evaluated imaging-detected vertebral, rib, and pelvic fractures, which are not an exact match to the fracture subset studied in prior studies in lymphoma patients (symptomatic osteoporotic fracture including vertebral and rib [14], imaging-detected vertebral fractures [15], any fracture identified by diagnosis codes [13]) and population studies of fracture rates in general populations (which primarily focus on vertebral fractures only in people without prevalent fracture). Thus, while we cite fracture rates in these studies to provide context and a point of reference for our findings, comparisons are not meant to be exact and should be interpreted with the differences in studies in mind. The high number of fractures identified in our study may be higher than reported in population studies of fractures for 2 reasons: (1) we included nonvertebral fractures such as rib and pelvic fractures, and (2) we used CT images which may be more sensitive than the plain radiographs used in most population studies, raising the possibility of detection bias. However, even if detection bias may inflate the number of incident vertebral fractures, it is unlikely to account for an increase from 0.5% (annual vertebral fracture incidence in women aged 65–69) [22] to 7% (our vertebral fracture incidence rate). Lastly, while we specifically looked for documented traumatic fractures in our chart review and excluded them in our analysis, the accuracy of this information is limited by the quality and completeness of documentation, as is the nature of retrospective chart reviews.

## Conclusions

Overall, our findings suggest that patients with aggressive B-cell NHL who receive R-CHOP-like therapy, particularly older patients, should be screened for fractures at baseline prior to treatment, and those with existing fractures should be considered for bone-directed therapy to further decrease risk of new fractures. Bone screening can be as simple as asking radiologists to review baseline scout and CT scans for signs of fracture or obtaining pre-treatment bone density studies. Treatment-related bone loss is well-recognized in patients with breast or prostate cancer due to chemotherapy-induced or hormone therapy-induced hypogonadism, and clear management guidelines have been developed [36, 37]. Similarly, guidelines also exist for bone health with steroid use for rheumatologic disorders [31]. However, there are no guidelines for the management of bone health in NHL patients despite the evidence showing treatment-related adverse effects and the existence of effective preventative therapies [38].

The ability to better identify those who are at highest risk of developing new fractures may help promote the use of bisphosphonates and other osteoporosis specific medications for reducing the risk of fractures in patients with aggressive B-cell NHL. In the future, we aim to derive a prediction score for identifying patients at high risk of developing incident fractures who may benefit from preventative therapies using clinical characteristics and CT-derived measures of bone mineral density.

## Data Availability

The authors had full control of all of the primary data and will allow the journal to review the data if requested.
